# Supplementary material for: The Kinetics of Lymphatic Dysfunction and Leukocyte Expansion in the Draining Lymph Node during LTB4 Antagonism in a Mouse Model of Lymphedema
Source: Int J Mol Sci. 2021 Apr 24;22(9):4455. doi: 10.3390/ijms22094455 (PMC8123113; doi:10.3390/ijms22094455)
Supplement: Supplementary file 1 [file ijms-22-04455-s001.zip › ijms-1150006-supplementary.pdf]

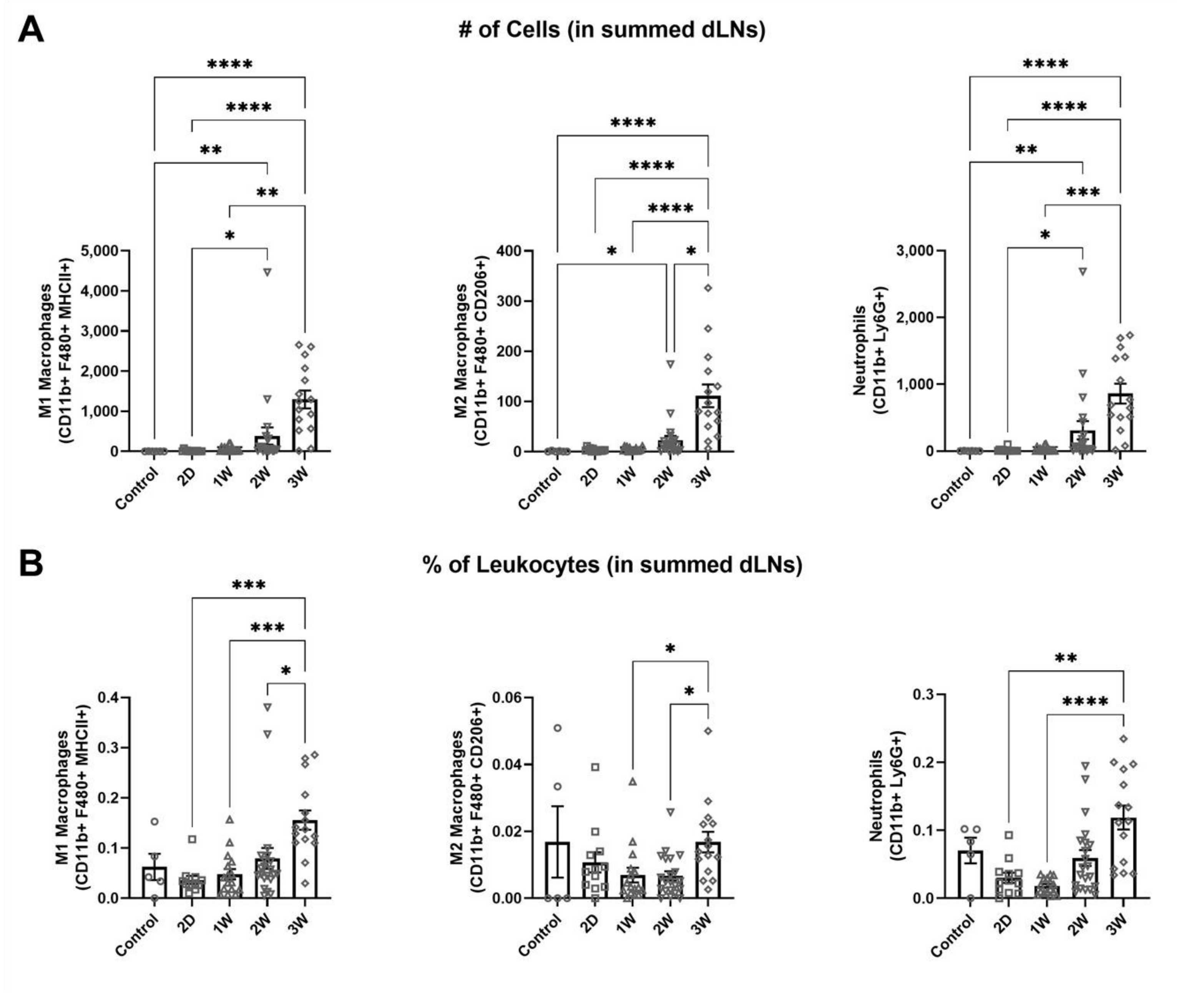

Supplemental Figure S1

M1 and M2 macrophage and neutrophil populations increase in dLNs during lymphedema progression. **(A)** Number of M1 macrophages, M2 macrophages, and neutrophils in dLNs for an unoperated control and after single vessel ligation lymphedema surgery at 2D, 1W, 2W, and 3W timepoints. Represented as the sum of the two dLNs. **(B)** M1 macrophage, M2 macrophage, and neutrophil frequencies of total leukocytes within dLNs for an unoperated control and after single vessel ligation lymphedema surgery at 2D, 1W, 2W, and 3W timepoints. Represented as the sum of the two dLNs for each leukocyte type divided by the sum of total leukocytes within the two dLNs. Kruskal-Wallis tests with Dunn's multiple comparisons were used to compare between timepoints for both number of cells and percentage of leukocytes. Control ($n = 6$), 2D ($n = 14$), 1W ($n = 16$), 2W ($n = 21$), 3W ($n = 15$). Mean ± s.e.m. * ($p < 0.05$), ** ($p < 0.01$), *** ($p < 0.001$), **** ($p < 0.0001$).

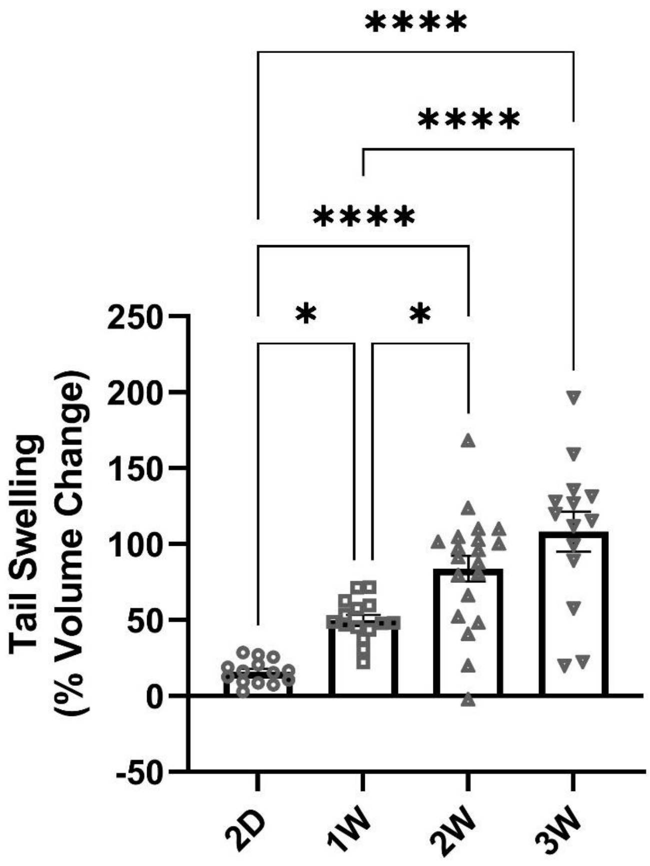

Supplemental Figure S2

Tail swelling following single vessel ligation surgery. Tail swelling following single vessel ligation surgery measured as percent change in volume from presurgery baseline. Swelling was measured at endpoint, specifically at 2D, 1W, 2W, and 3W timepoints. One-way ANOVA tests with Tukey's multiple comparisons were used to compare between timepoints. Mean ± s.e.m. * ($p < 0.05$), **** ($p < 0.0001$).

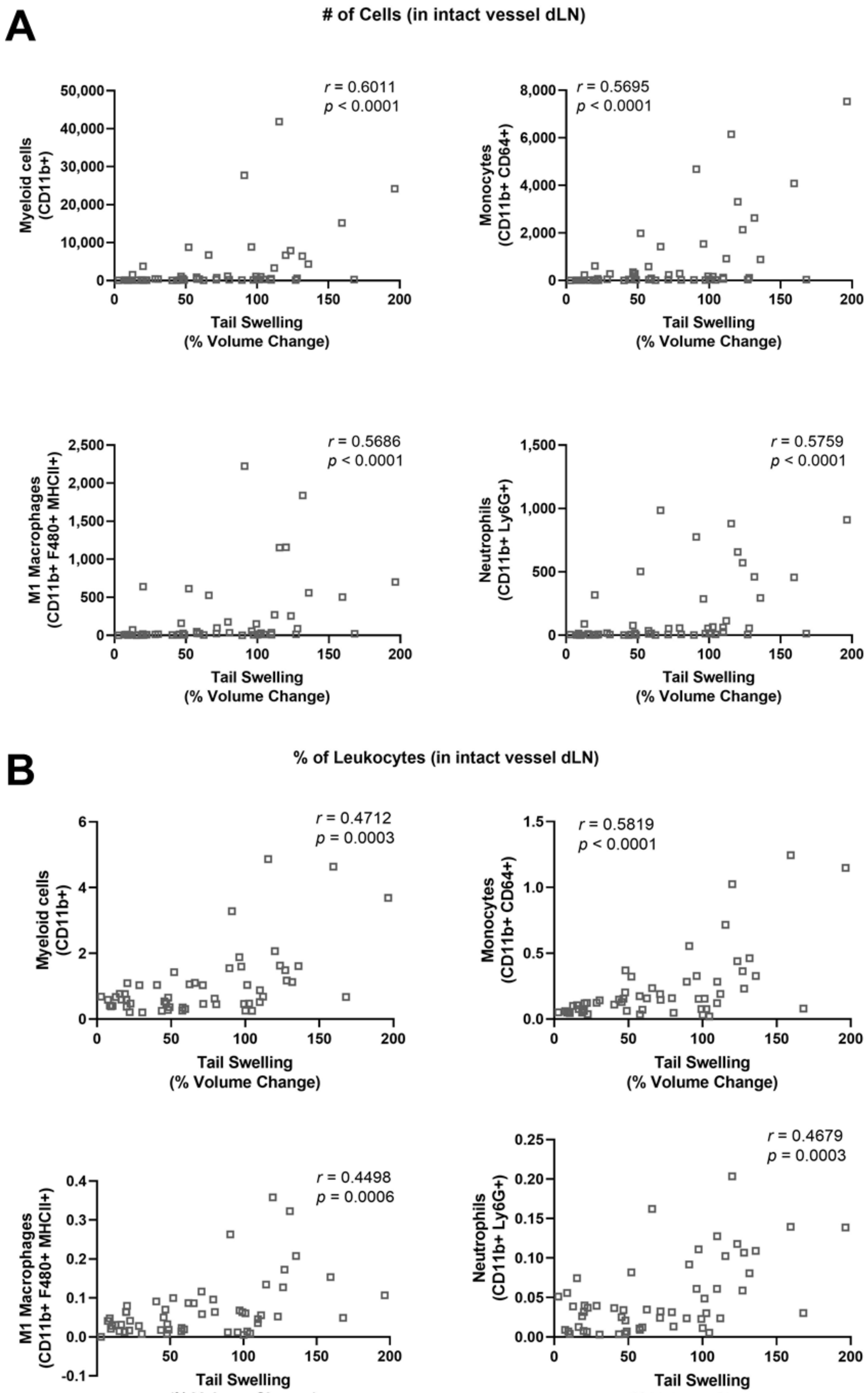

Supplemental Figure S3

Myeloid cell expansion in dLNs correlates with increase in tail volume after lymphedema surgery. **(A)** Spearman's rank correlation between number of cells in intact vessel dLN and percent volume change following single vessel ligation lymphedema surgery for myeloid cells, monocytes, M1 macrophages, and neutrophils. **(B)** Spearman's rank correlation between fraction of leukocytes in intact vessel dLN and percent volume change following single vessel ligation lymphedema surgery for myeloid cells, monocytes, M1 macrophages, and neutrophils.

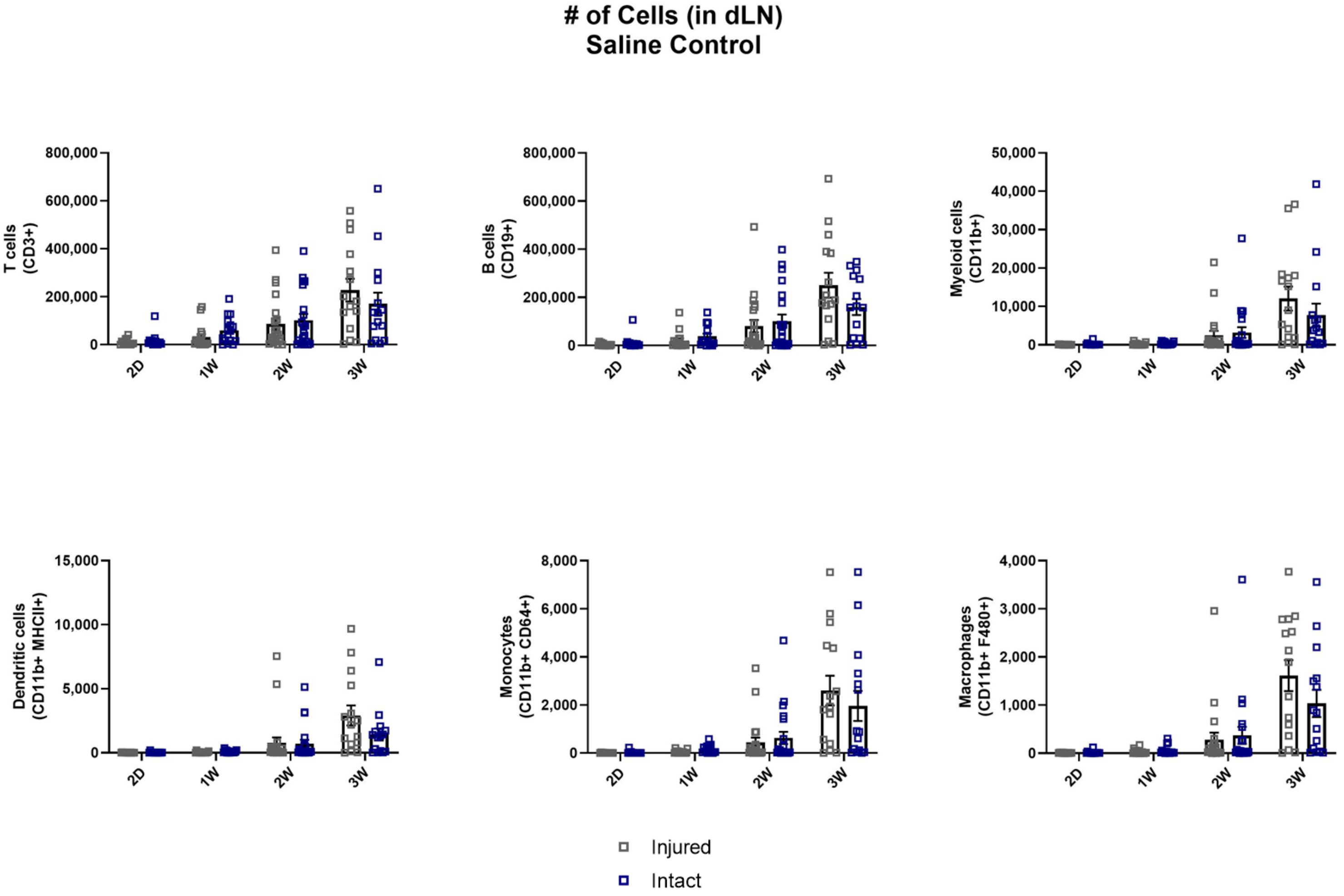

Supplemental Figure S4

Leukocyte expansion in intact versus injured vessel dLNs during lymphedema progression. Number of T cells, B cells, CD11b+ cells, dendritic cells, monocytes, and macrophages in both injured and intact vessel dLNs after single vessel ligation lymphedema surgery at 2D, 1W, 2W, and 3W timepoints. Multiple Mann-Whitney tests using the Holm-Šídák method were used to compare between injured and intact vessel dLNs at each timepoint. Mean ± s.e.m.

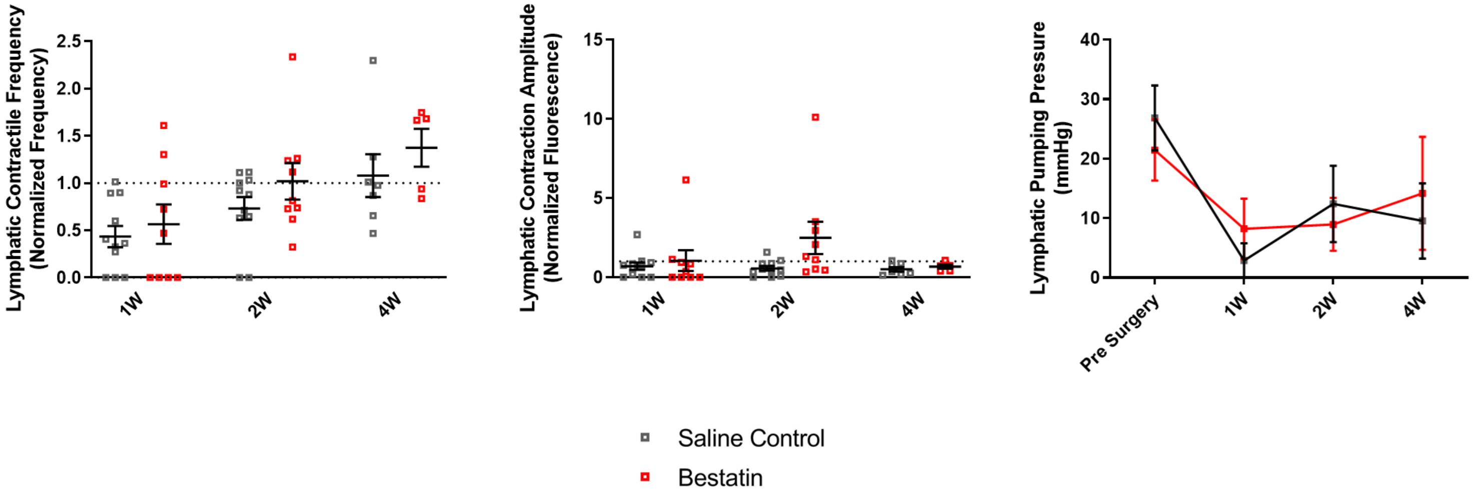

Supplemental Figure S5

Bestatin treatment shows no effect on various metrics of lymphatic pumping function measured through NIR imaging. Quantification of lymphatic contractile frequency and amplitude normalized to presurgery baseline in both bestatin-treated and saline control groups at 1W, 2W, and 4W timepoints. Pumping pressure in the intact vessel is also quantified at the same timepoints. A mixed-effects model with Šídák's multiple comparisons was used to compare between treatments for each timepoint. Saline Control: 1W (n = 11), 2W (n = 11), 4W (n = 7); Bestatin: 1W (n = 9), 2W (n = 9), 4W (n = 5). Mean ± s.e.m.

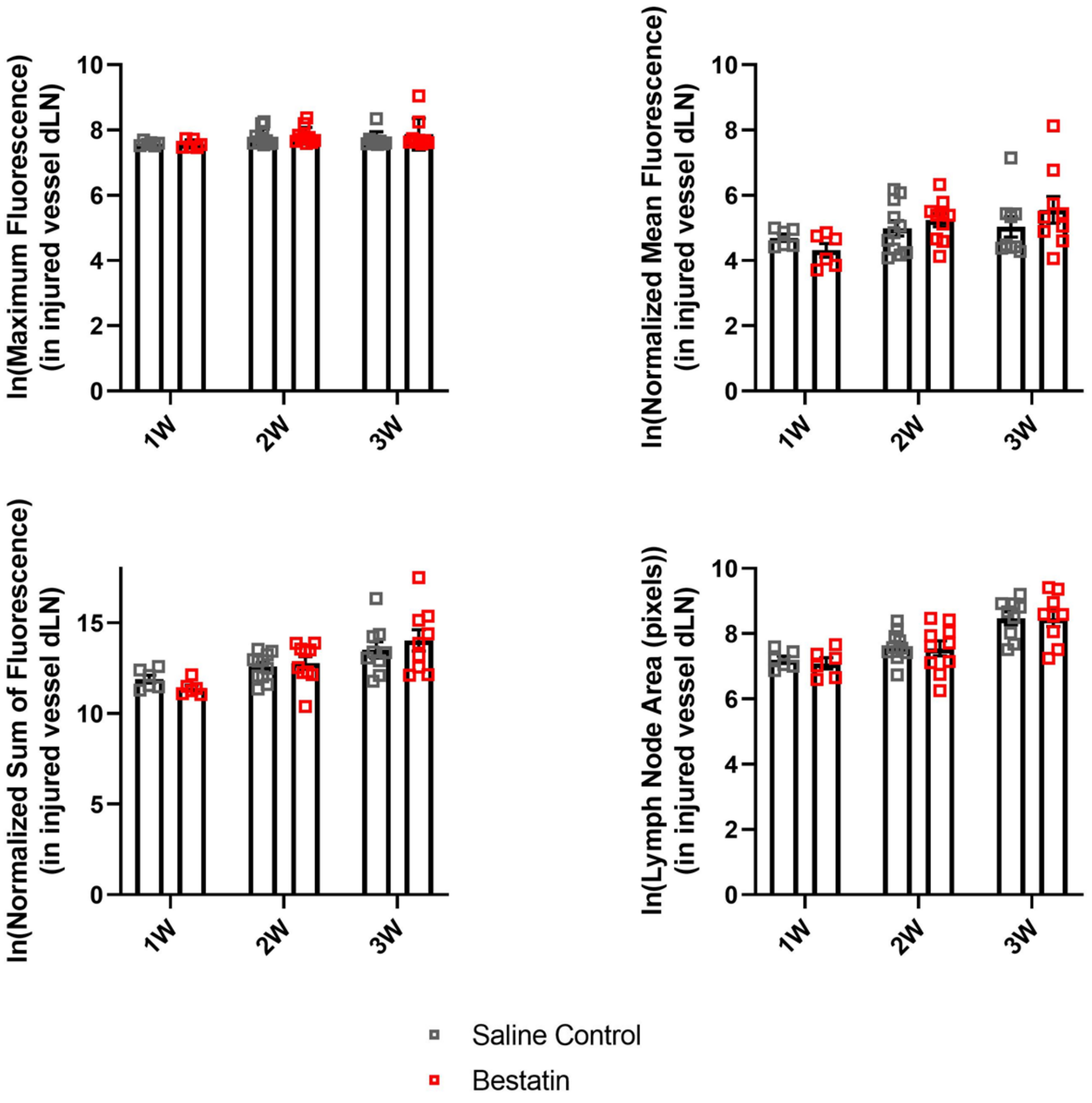

Supplemental Figure S6

Bestatin treatment does not change particle uptake to the injured vessel dLN during lymphedema progression. Natural logarithm of maximum fluorescence, normalized mean fluorescence, normalized sum of fluorescence, and lymph node area in the injured vessel dLN for the saline control group and the bestatin-treated group at 1W, 2W, and 3W timepoints. Two-way ANOVA with Šídák's multiple comparisons to compare between treatments at each timepoint. 1W (n = 6), 2W (n = 11), 3W (n = 9). Mean ± s.e.m.

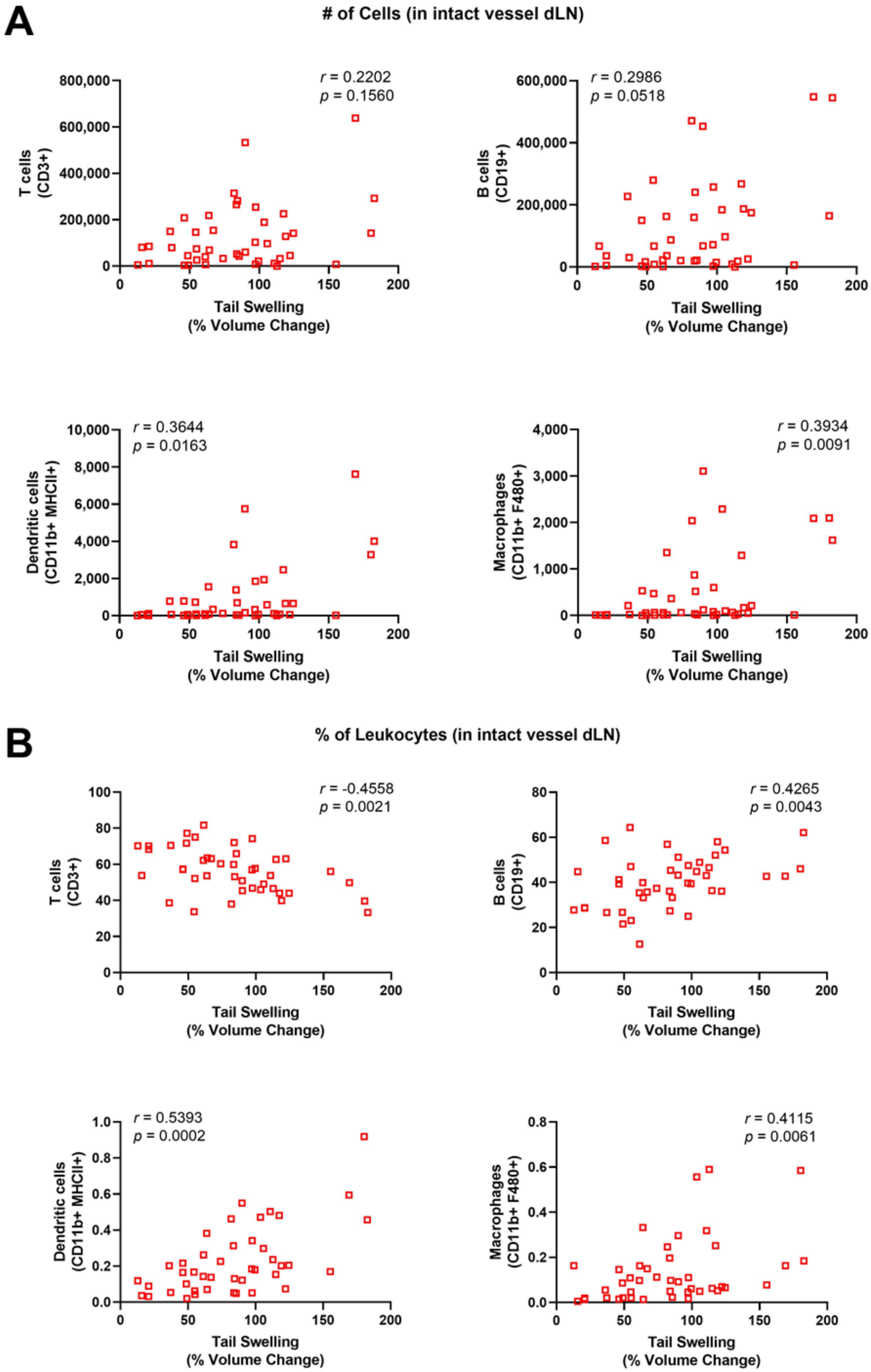

Supplemental Figure S7

Leukocyte expansion in dLNs following bestatin treatment correlates with increase in tail volume after lymphedema surgery. **(A)** Spearman's rank correlation between number of cells in intact vessel dLN and percent volume change following single vessel ligation lymphedema surgery for T cells, B cells, dendritic cells, and macrophages. **(B)** Spearman's rank correlation between fraction of leukocytes in intact vessel dLN and percent volume change following single vessel ligation lymphedema surgery for T cells, B cells, dendritic cells, and macrophages. All data shown is from bestatin-treated mice.

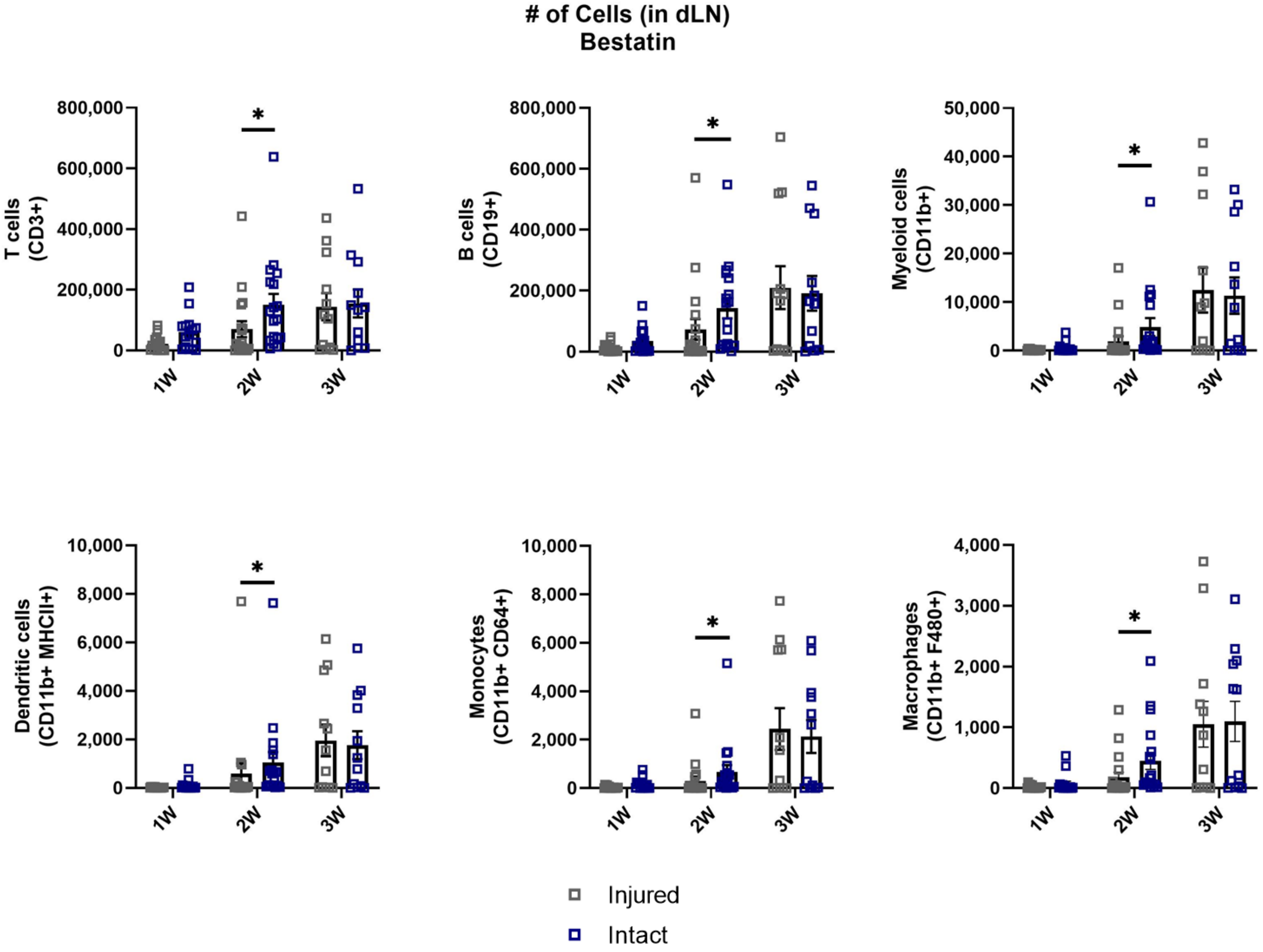

Supplemental Figure S8

Leukocyte expansion in intact versus injured vessel dLNs following bestatin treatment during lymphedema progression. Number of T cells, B cells, CD11b+ cells, dendritic cells, monocytes, and macrophages in both injured and intact vessel dLNs following single vessel ligation lymphedema surgery and bestatin treatment at 1W, 2W, and 3W timepoints. Multiple Mann-Whitney tests using the Holm-Šídák method were used to compare between injured and intact vessel dLNs at each timepoint. Mean ± s.e.m. * ($p < 0.05$).

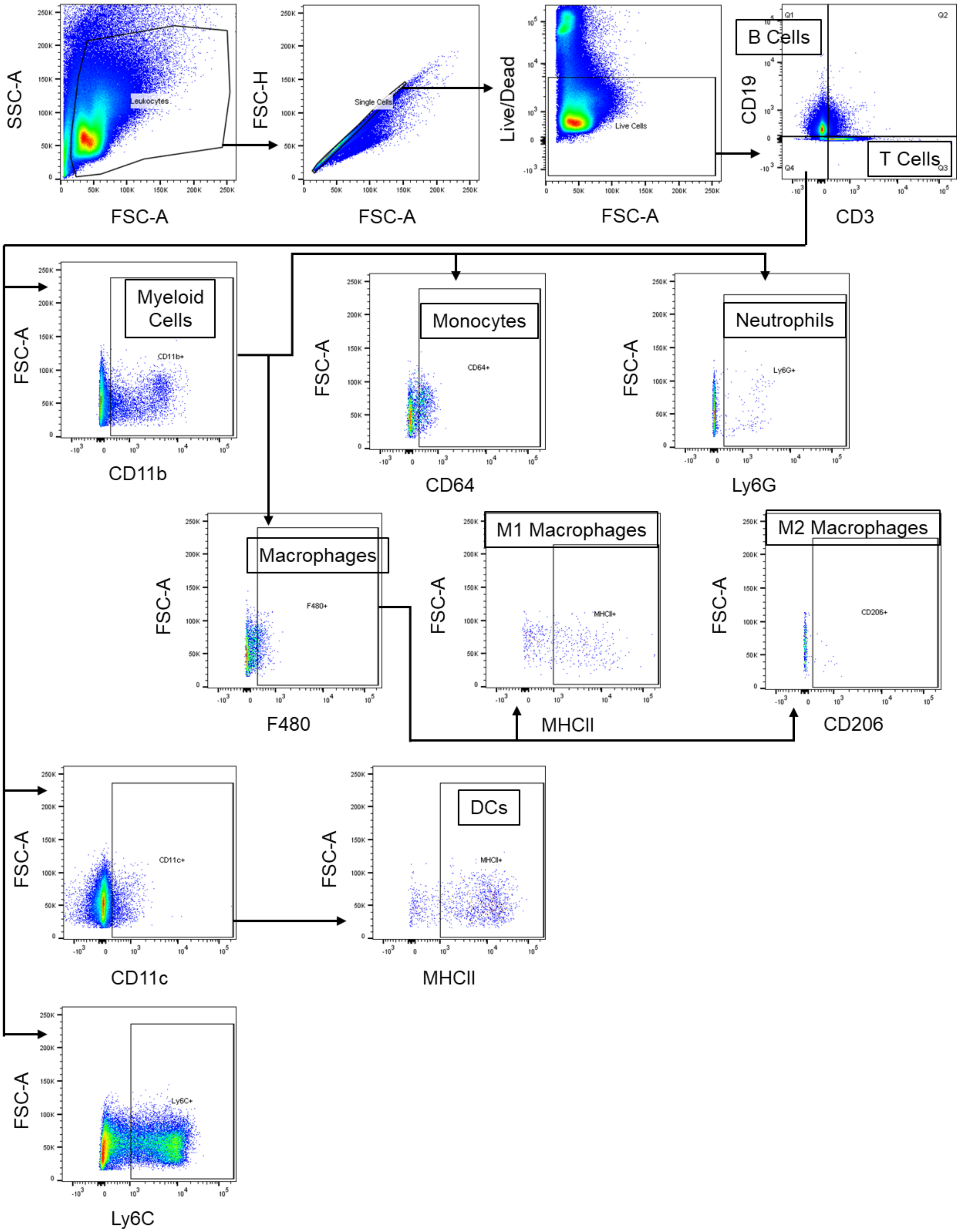

Supplemental Figure S9

Gating strategy for flow cytometry analysis. The gating strategy to classify the following leukocyte subsets: T Cells (CD3+), B Cells (CD19+), Myeloid cells (CD11b+), Dendritic cells (CD11c+ MHCII+), Monocytes (CD11b+ CD64+), Macrophages (CD11b+ F480+) including M1 (MHCII+) and M2 (CD206+) polarization, and Neutrophils (CD11b+ Ly6G+).

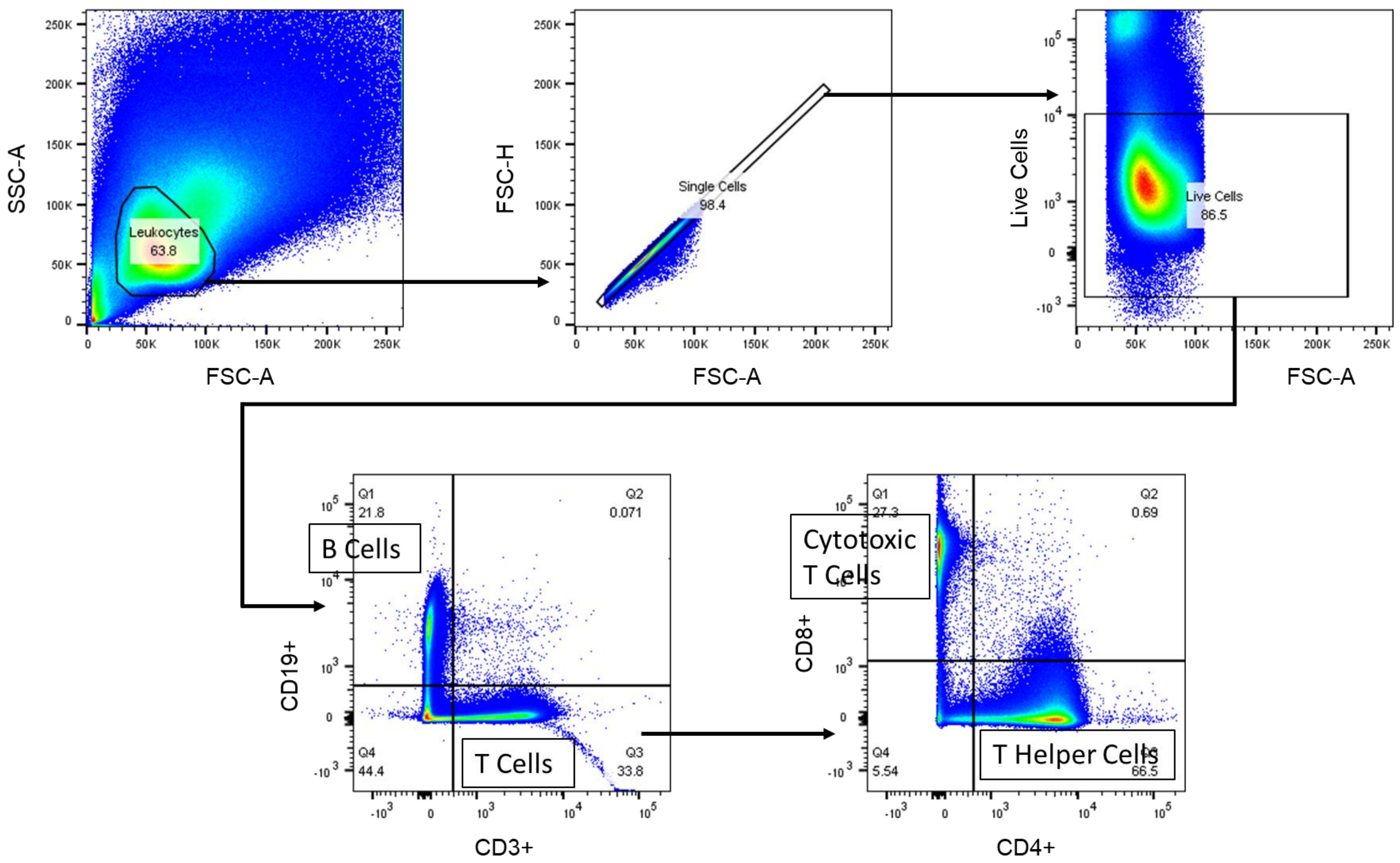

Supplemental Figure S10

Gating strategy for flow cytometry analysis of T cell subpopulations. The gating strategy to classify T helper cells (CD3+ CD4+) and cytotoxic T cells (CD3+ CD8+).

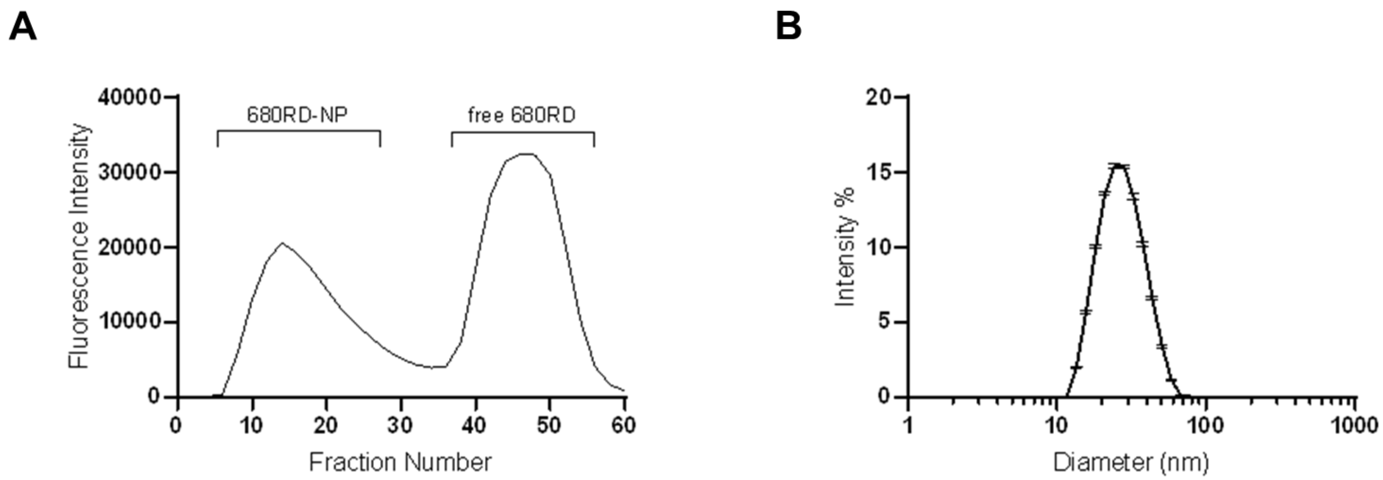

Supplemental Figure S11

Characterization of nanoparticles labeled with IRdye 680RD (680RD-NP). **(A)** Elution profile of 680RD-NP from a CL-6B size exclusion chromatography column. Separate peaks for 680RD-NP and unconjugated 680RD clearly emerge, allowing for cleaning and isolation of NP fractions. **(B)** NP diameter, measured by dynamic light scattering.
